# Social Disparities in Lifestyle and Body Weight Changes during COVID-19 Quarantine and Post-Quarantine Persistence of Changes among Lithuanian Adult Population

**DOI:** 10.3390/nu15194254

**Published:** 2023-10-04

**Authors:** Janina Petkevičienė, Asta Raskilienė, Monika Grincaitė, Vilma Kriaučionienė

**Affiliations:** 1 Health Research Institute, Faculty of Public Health, Lithuanian University of Health Sciences, Tilzes Str. 18, 47181 Kaunas, Lithuania; 2 Department of Preventive Medicine, Faculty of Public Health, Lithuanian University of Health Sciences, Tilzes Str. 18, 47181 Kaunas, Lithuania; 3 Institute of Biology Systems and Genetic Research, Lithuanian University of Health Sciences, Eiveniu Str. 4, 50103 Kaunas, Lithuania

**Keywords:** COVID-19, adults, eating habits, physical activity, body weight, post-quarantine period, changes

## Abstract

The role of social factors on changes in lifestyle habits during the COVID-19 quarantine and their maintenance post-quarantine is underexamined. This study aimed to assess sociodemographic differences in nutrition, physical activity and body weight changes during the quarantine and their persistence post quarantine. The study included a random sample of Lithuanian residents aged 20 to 64. The questionnaires were filled in during the face-to-face interview within households countrywide in 2023. In total, 1500 individuals (742 men and 758 women) participated in the survey. Up to 34.9% of the respondents reported changes in eating habits, mostly increased consumption, 29.5% indicated reduced physical activity, and 22.7% gained weight. Multivariate logistic regression analysis revealed that highly educated individuals were more likely than poorly educated individuals to increase healthy and decrease unhealthy food consumption and increase physical activity during the quarantine. The city inhabitants reported unfavourable changes in nutrition habits more often than those living in villages did. The proportion of respondents who reported the maintenance of the quarantine changes in lifestyle depending on the habit varied from 23.2% to 71.4% in the post-quarantine period. Highly educated individuals were less likely to maintain the decreased consumption of fresh fruits and increased body weight than less-educated individuals were. Our study provides evidence that the targeted interventions are needed to help less-educated individuals and city residents develop and maintain healthy lifestyle habits, even in times of crisis.

## 1. Introduction

Social inequalities in nutrition habits, physical activity and body weight, potentially exacerbating health inequities, have been reported in many studies [1,2]. People of lower educational attainment and with a lower socio-economic status were most likely to consume unhealthy foods, to be less physically active and to have overweight or obesity [3,4].

The COVID-19 pandemic disrupted the daily life of individuals across all strata of society. Governments in most countries imposed restrictions to limit the spread of the infection. In Lithuania, the quarantine lasted from 16 March 2020 until 1 July 2021 [5]. Later, a state-level situation of emergency was declared, which was lifted on 1 May 2022. Throughout the quarantine period, strict measures were implemented, including limitations on travelling within the country, the closure of public institutions and numerous commercial and dining establishments, a shift to remote learning for educational institutions, the restriction of activities of health care institutions, and the prohibition of mass events and gatherings. These restrictions varied over time in their degree of intensity. Under these novel circumstances, maintaining a balanced diet and regular physical activity posed a considerable challenge. People had less access to shops and were more dependent on highly processed food. Moving restrictions and the closure of gyms reduced the physical activity possibilities. People stayed at home and adopted sedentary behaviours.

The impact of the COVID-19 pandemic on diet, physical activity and body weight in the general population has been extensively studied worldwide [6,7,8]. Most studies revealed the adverse effect of restrictions on nutrition habits, including the increased consumption of unhealthy food such as fast food, ultra-processed items and sweets, frequent snacking between meals and greater overall food intake [9,10,11]. Conversely, in some countries, the lockdown led to better adherence to the Mediterranean diet and higher vegetable consumption [12,13,14]. Physical activity decreased in most populations [15,16]. Unhealthy changes in nutrition and physical activity are associated with weight gain [17,18]. The restrictive measures, uncertainty and anxiety caused by the pandemic disrupted the usual routines, reduced social contact, and worsened people’s mental well-being, leading to sleep disturbances and increased alcohol, nicotine, and drug use in some populations [9,19,20,21].

A previous study conducted in Lithuania during the strict quarantine showed an increase in snacking, the consumption of home-made pastries and fried food and a decrease in fast food, commercial pastry and sweetened drink consumption [22]. These shifts in dietary habits, together with reduced physical activity, were related to weight gain in nearly a third of the participants.

The effect of the COVID-19 pandemic on lifestyle has predominantly been examined in the whole population, rarely evaluating the influence of social factors. Understanding which social groups were most affected by the pandemic is crucial for targeted interventions to prevent unhealthy lifestyle habits. Only a few studies analysed the sociodemographic differences in changes in eating and physical activity habits during the COVID-19 quarantine [23,24,25]. Furthermore, there is a significant lack of data on how long the changes in lifestyle habits of different social groups persisted in the post-quarantine period.

To better understand the impact of the quarantine on the lifestyle of different social groups and the long-term health effects, this study aimed to examine sociodemographic differences in the changes in nutrition habits, physical activity and body weight during the COVID-19 quarantine and the persistence of these changes in the post-quarantine period in the Lithuanian adult population.

## 2. Materials and Methods

### 2.1. Study Design and Sample

Face-to-face interviews were conducted within households between 5th and 18th January 2023. Data were collected by an independent institution of public opinion and market research ‘Vilmorus Ltd.’. The participants of this study were Lithuanian residents aged from 20 to 64 selected using a multi-stage, probabilistic and proportional sampling technique. This method ensured equal opportunities for all Lithuanian residents, representing different places of residence and social groups, to participate in the study. The sample had to correspond to the demographic distribution of the Lithuanian population in terms of age, gender and place of residence.

The survey was conducted in all ten administrative regions (counties) of Lithuania. The number of individuals interviewed in each county was proportional to its population size, as established using national statistical data. Within each county, the county centre or another town and rural localities were chosen through random selection. Next, streets and the starting point of the route were randomly selected in each location. The interviewer visited every sixth house or apartment. To ensure a representative respondent within each household, the ‘last birthday rule’ was applied. If the intended respondent was unavailable, a subsequent visit was arranged. The survey was carried out in 27 cities and over 40 villages by a team of 69 professional interviewers. Additionally, to enhance the comprehensiveness of the data collection, every tenth respondent was contacted via phone to inquire further about the survey.

The study protocol was approved by the Bioethics Centre at the Lithuanian University of Health Sciences (protocol No. BEC-GM(M)-106). All the participants were informed about the study. Participation was voluntary and anonymous. Verbal consent to participate in the study was received from all the participants.

### 2.2. Questionnaire and Variables

The study questionnaire included questions about sociodemographic characteristics: sex, age, education and place of residence. The respondents were grouped into the following categories according to educational attainment: (1) low education level (primary education, incomplete secondary education, secondary school or vocational school) and (2) high education level (college or university). According to the administrative classification of places of residence, the respondents were categorized as living in cities (the capital city and four largest cities of Lithuania), towns (centres of municipalities or smaller towns and villages.

The respondents were asked to report about changes in the listed lifestyle habits and body weight during the COVID-19 quarantine compared to those during the pre-quarantine period. The response choices were: ‘Increased’, ‘Remained unchanged’ and ‘Decreased’. Next, the respondents who reported changes were asked: ‘Post-quarantine, did your lifestyle habits and body weight revert to the pre-quarantine level?’ The response options for each lifestyle habit and body weight were: ‘Changes persisted’ and ‘Reverted to pre-quarantine levels.’

A food frequency questionnaire was used to assess nutrition habits after the COVID-19 quarantine. Sixteen food groups were included in the questionnaire. For data analysis, the respondents were categorized into three groups by food consumption frequency: (1) ‘daily consumption’ (‘several times a day’ or ‘daily’), (2) several times a week, and (3) 1–4 times a month or less frequently (‘1–4 times a month’ or ‘never’).

Physical activity was evaluated by asking: ‘In your leisure time, how often do you do physical exercise for at least 30 min, which makes you at least mildly short of breath or perspire?’. The participants were grouped into those who were physically active at least four times a week (answers ‘daily’ and ‘4–6 times a week’) or less often.

The respondents were asked to indicate their height and weight. Their BMI was calculated by dividing the weight (in kilogrammes) by the square of the height (in meters). Based on BMI values, the participants were categorized as underweight (BMI < 18.5 kg/m^2^), normal weight (BMI 18.5–24.9 kg/m^2^), overweight (BMI 25–29.9 kg/m^2^) or obese (BMI > 30 kg/m^2^).

### 2.3. Statistical Analysis

Categorical variables are expressed as percentages. Multivariate logistic regression analysis was employed to assess the associations of nutrition habits, physical activity and weight after the COVID-19 quarantine with education and place of residence. The associations of changes in lifestyle habits and body weight during the COVID-19 quarantine and the persistence of changes in the post-quarantine period with sociodemographic variables such as education and place of residence were also evaluated using multivariate logistic regression analysis. The same analysis was performed to assess the associations between changes in lifestyle habits and body weight during the COVID-19 quarantine and the characteristics of the same variables in the subsequent post-quarantine period. Sex and age were included as covariates in all the models. In all the analyses, separate logistic regression models were generated for every food item, physical activity and weight. The criterion for statistical hypothesis testing was established at a significance level of 0.05.

Data analysis was conducted using the IBM SPSS Statistics software package, version 29.0 (IBM Corp.: Armonk, NY, USA, released 2022).

## 3. Results

In total, 1500 individuals (742 men and 758 women) participated in the survey. The characteristics of the participants are presented in Table 1.

Some participants responded that they had changed their nutrition habits during the COVID-19 quarantine. The consumption of home-cooked food, the frequency of snacking and the quantity of food eaten increased the most (Table 2). Almost every tenth respondent increased their consumption of home-made confectionery, fast food and home-delivered food. The proportion of participants who answered that they reduced the consumption of some foods was lower. The decrease in consumption of commercial confectionery and home-delivered food from catering establishments was reported most often. Even 29.5% of respondents reduced their physical activity during the quarantine. An increase in body weight was reported by 22.7% of participants.

Multivariate logistic regression analysis revealed that the participants with university or college education were more likely to increase their consumption of healthy foods such as fish, cereals, fresh vegetables and fruits than the individuals with a lower education level were (Table 3). Highly educated respondents ordered food from stores during the quarantine more often than poorly educated people did. Furthermore, they had 2.45 times higher odds of increased physical activity. The likelihood of the increase in unhealthy food consumption, such as commercial confectionery, home-made confectionery and fast food, was higher in cities compared to that in villages. The city inhabitants increased their frequency of snacking and consumed home-cooked and home-delivered food more often than those living in villages did. The changes in body weight did not depend on education and place of residence.

A high education level was associated with higher odds of reduction in unhealthy food consumption, such as home-made confectionery, chocolate and sweets, sweetened drinks and fast food consumption (Table 4). The city inhabitants were less likely to decrease their consumption of meat and meat products, fresh fruits, commercial and home-made confectionery, chocolate and sweets and fast food than those living in villages were. The odds of decreased body weight were lower among the city inhabitants than the village inhabitants.

Changes in eating habits, physical activity and body weight during the COVID-19 quarantine were associated with post-quarantine lifestyle and weight (Table 5). The likelihood of the increase in healthy and unhealthy food consumption rose with a higher frequency of consumption of the same foods in the subsequent post-quarantine period. Physical activity increased during the quarantine most among the participants who exercised four days a week or more often in their leisure time. The odds of the increase in body weight were 2.74 times higher among the respondents with overweight and 3.05 times higher among those with obesity than they were among the individuals with a low or normal body weight.

The participants who consume fruits and vegetables daily were less likely to reduce the consumption of those healthy foods during the quarantine (Table 5). However, the individuals who consumed commercial confectionery, chocolate, sweets and sweetened drinks daily were also less likely to decrease their consumption during the quarantine. The odds of reduced physical activity during the quarantine were not statistically significant among the respondents who exercised four days a week or more often compared to those who were involved in leisure time physical activity less often than once a week. The decrease in body weight during the quarantine was not associated with the weight in the post-quarantine period.

Some changes in lifestyle habits and body weight that occurred during the COVID-19 quarantine persisted after the quarantine (Table 6). More than 60% of respondents who indicated they increased their consumption of fish and seafood, fresh vegetables, fruits or berries and cereal products during the quarantine maintained these habits in the post-quarantine period. Even 40.9% of participants maintained the increased habit of ordering food from stores. More than half (54.3%) of the respondents who increased their physical activity during the quarantine exercised more often after the quarantine as well. Unfortunately, weight gain during the quarantine persisted among 64.3% of the participants.

A significant number of individuals who reduced their unhealthy eating habits during the quarantine, such as the consumption of confectionery, sweets, sweetened drinks and fast food, continued to eat healthier after the quarantine. Decreased physical activity persisted among 30.0% of the participants.

The persistence of a few changes in lifestyle habits in the post-quarantine period was associated with education and place of residence (Table 7). Highly educated individuals were less likely to maintain the decreased consumption of fresh fruits and the amount of food eaten as well as an increased body weight. The odds of the persistence of increased vegetables and home-made confectionary consumption and reduced physical activity were lower among city than those of the village inhabitants.

The characteristic of the respondents’ lifestyle habits and body weight in the post-quarantine period are presented in Table 8. Only slightly more than half of the respondents stated that they consume fresh vegetables and fruits daily, 59.1% and 50.4%, respectively. Every third participant consumed legumes, and every second one consumed nuts at least a few days a week. Cereal products were consumed daily by 22.9% of the respondents. About half of the participants (51.3%) reported consuming red meat a few days a week, and 25.8% consumed it daily. Almost every second respondent consumed confectionery, chocolate and sweets a few days a week or daily. Only 27.8% of the participants were involved in leisure time moderate physical activity four or more days a week. The prevalence of overweight was 39.0%, and the prevalence of obesity was 13.4%.

Nutrition habits after the COVID-19 quarantine were associated with the education of the respondents (Table 9). Individuals with university or college education were more likely to consume healthy foods such as fresh fruits, vegetables and porridges daily than the less-educated participants were. Higher odds of fish, legume and nut consumption at least several times a week were also found for the highly educated individuals. Meanwhile, the odds of unhealthy food, such as red meat and meat products, sweetened drinks, fast food and snacks, consumption were lower among the highly educated respondents. The participants with university or college education were less likely to be overweight than the less-educated people were.

The nutrition habits of the city inhabitants were healthier compared to those living in villages (Table 2). The city residents consumed fruits, vegetables, nuts and fish more often, while they consumed sweetened drinks less frequently than the village inhabitants did. The town residents were less likely to consume confectionery, sweetened drinks, fast food and unhealthy snacks than those living in villages were. On the other hand, the odds of being physically active four days a week were lower for the town dwellers than they were for the village inhabitants. The likelihood of being overweight was lower for the city than it was for the village residents.

## 4. Discussion

In this study, we examined the changes in nutrition habits, physical activity and body weight according to the education and place of residence of the Lithuanian adult population during the COVID-19 quarantine and the persistence of these changes in the subsequent post-quarantine period. A substantial number of the participants reported that they changed their nutrition and physical activity habits during the quarantine, mostly increasing their food intake and decreasing their physical activity level. Nearly one fifth of the participants indicated weight gain. A significant proportion of the individuals who changed their lifestyle habits and body weight during the quarantine indicated the persistence of these changes post quarantine. Education and place of residence were associated with changes in diet and physical activity during the quarantine and maintenance of these changes post quarantine.

Our study found that Lithuanian adults with a higher educational attainment and those living in cities had healthier lifestyle habits and a lower prevalence of overweight after the COVID-19 quarantine than less-educated and village residents did. The previous studies carried out in Lithuania demonstrated an improvement in the nutrition habits of the adult population during recent decades [26]. The frequency of the consumption of fresh vegetables and the intake of healthy fats increased, while the consumption of saturated fats decreased. The most favourable changes in the diet were observed in the highly educated individuals and city residents.

The changes in lifestyle habits reported by the respondents of our study were similar to those found in previous studies [9,10,23,24]. We determined that highly educated individuals were more likely to change their lifestyle in a health-promoting direction than those with a poor education were. Meanwhile, the city residents have changed their diet in an unfavourable direction, consuming more unhealthy foods during the quarantine. A limited number of studies have been undertaken to examine variations in these lifestyle changes across different social groups during the COVID-19 pandemic. In a study carried out in Greece, 36.4% of the participants reported that they changed their dietary habits during the pandemic towards a healthier diet. Among those, 69% had a higher education degree [27]. A study conducted in the Netherlands demonstrated that the participants with a high education level more frequently reported eating less healthy food and purchasing more sweet snacks (pastries and chocolate) during lockdown compared to those with a low education level [23]. The highest increase in the Healthy Eating Index during the lockdown was shown among the less-educated participants in a Canadian study [28]. Favourable changes in food consumption induced by the COVID-19 pandemic were more prominent for Chinese residents who lived in rural areas than urban areas [29]. A Spanish study found that people who lived in rural areas during the quarantine were more physically active than those who lived in urban areas were [30]. Thus, the data from studies on social differences in lifestyle changes during the pandemic are conflicting. Further research is needed to assess the implications of these changes for social disparities in lifestyle and health post pandemic.

In our study, 22.7% of the participants indicated weight gain during the quarantine. We did not find any association between weight gain and education as well as place of residence. Similarly, no association between education and weight gain was observed in a study carried out in Japan [31]. A study conducted in the United States found that weight gainers were more likely to have a high school education and live in rural areas [32].

Our study revealed strong links between the changes during the quarantine and lifestyle habits after the quarantine. To the best of our knowledge, only a few studies provided data about nutrition habits during and after the COVID-19 quarantine. A Danish study reported that after the lockdown, some favourable changes in diet, including reduced energy intake and decreased intake of saturated fat and added sugars, were noticed [22]. However, the intake of dietary fibre, whole grain and fish decreased, and the intake of red meat increased. Educational attainment had a significant impact on the change in dietary fibre intake, as those with upper secondary school and higher education or those holding a Ph.D. showed a reduction in fibre intake following the lockdown.

One of the key drivers of social inequalities in nutrition habits during the pandemic is uneven access to healthy foods. Low-income individuals often face barriers to obtaining fresh foods due to financial constraints. As lockdowns and movement restrictions were imposed, these groups were more likely to rely on cheaper, processed and calorie-dense foods, leading to imbalanced diets with lower nutritional quality. Education plays a crucial role in increasing individuals’ ability to make informed dietary choices. The lack of evidence-based knowledge about healthy nutrition can maintain unhealthy eating patterns and prevent attaining a balanced diet. Disparities in education, income and access to healthy food can contribute to divergent dietary patterns, potentially exacerbating health inequities.

The strengths of this study include the large random sample, which is representative of the general Lithuanian adult population regarding sex, age, education and place of residence; countrywide data collection; the use of face-to-face interviews; the short interval between the end of the COVID-19 quarantine and the beginning of the survey; and the assessment of the persistence of quarantine changes in lifestyle and body weight in the subsequent post-quarantine period. Also, our findings increase the limited knowledge available regarding sociodemographic differences in changes in eating habits and physical activity during and after the COVID-19 quarantine.

This study has some limitations. First, all the collected data were self-reported, which can affect the accuracy and reliability of the information gathered. The respondents might have provided answers that are socially acceptable rather than accurate. They may underreport behaviours that are seen as negative or overreport behaviours that are seen as positive. The accuracy of self-reported data depends on memory because people’s ability to recall past events or experiences can vary widely. BMI calculations were based on self-reported weight and height data, and potential inaccuracies in reporting should be considered. Second, our study lacked information about the pre-pandemic lifestyle habits and body weight of the respondents. Finally, the cross-sectional study design did not allow us to establish causal associations.

## 5. Conclusions

This study showed that the COVID-19 quarantine affected the nutrition habits, physical activity and body weight of Lithuanian adults. More people increased rather than decreased their food intake, which, combined with reduced physical activity, led to weight gain. Changes in diet and physical activity during the quarantine were associated with education and place of residence. Highly educated individuals were more likely to increase their consumption of healthy foods and increase their physical activity level, while the city inhabitants reported unhealthy changes in nutrition habits more often than those living in villages did. Many individuals who changed their lifestyle habits and body weight during the quarantine indicated the persistence of these changes post quarantine. Highly educated individuals were less likely to maintain the decreased consumption of fresh fruits and an increased body weight than the less-educated people were. Our study provided evidence that the targeted interventions are needed to help less-educated individuals and city residents develop and maintain healthy lifestyle habits, even in times of crisis. Future studies should focus on the long-term impact of the COVID-19 pandemic on the lifestyle and health of individuals.

## Figures and Tables

**Table 1:** Sociodemographic characteristics of the study population.

Characteristic	N	%
**Sex**		
Male	742	49.5
Female	758	50.5
**Age (years)**		
20–34	467	31.1
35–49	487	32.5
50–64	546	36.4
**Education**		
Low	998	66.5
High	502	33.5
**Place of residence**		
Cities	650	43.3
Towns	363	24.2
Villages	487	32.5

**Table 2:** Distribution of respondents according to lifestyle and body weight changes during the COVID-19 quarantine.

Variables	Changes					
	Increased		Unchanged		Decreased	
	N	%	N	%	N	%
**Food intake**						
Meat and meat products	101	6.7	1306	87.1	93	6.2
Fish and seafood	70	4.7	1323	88.2	107	7.1
Milk and milk products	87	5.8	1354	90.3	59	3.9
Cereal products	104	6.9	1352	90.1	44	2.9
Fresh vegetables	141	9.4	1323	88.2	36	2.4
Fresh fruits or berries	129	8.6	1317	87.8	54	3.6
Commercial confectionery	111	7.4	1208	80.5	181	12.1
Home-made confectionery	217	14.5	1180	78.7	103	6.9
Chocolate and sweets	148	9.9	1232	82.1	120	8.0
Sweetened drinks, lemonade	91	6.1	1268	84.5	141	9.4
Fast food	175	11.7	1181	78.7	144	9.6
**Eating habits**						
Quantity of food eaten	319	21.3	1086	72.4	95	6.3
Frequency of snacking	437	29.1	989	65.9	74	4.9
Home-cooked food	523	34.9	933	62.2	44	2.9
Home-delivered food from catering establishments	175	11.7	1167	77.8	158	10.5
Home-delivered food from stores	198	13.2	1160	77.3	142	9.5
**Physical activity**	131	8.7	927	61.8	442	29.5
**Body weight**	341	22.7	1068	71.2	91	6.1

**Table 3:** Odds ratios for increased lifestyle habits and body weight during the COVID-19 quarantine by education and place of residence.

Variables	Education *			Place of Residence **					
	High Versus Low			Towns Versus Villages			Cities Versus Villages		
	OR	95% CI	*p*-Value	OR	95% CI	*p*-Value	OR	95% CI	*p*-Value
**Increased food intake**									
Fish and seafood	2.05	1.23; 3.40	**0.006**	1.64	0.80; 3.36	0.174	1.70	0.89; 3.24	0.108
Cereal products	1.74	1.14; 2.67	**0.011**	1.12	0.65; 1.93	0.692	1.04	0.64; 1.70	0.872
Fresh vegetables	1.63	1.13; 2.36	**0.010**	0.89	0.54; 1.45	0.634	1.05	0.69; 1.59	0.832
Fresh fruits or berries	1.64	1.11; 2.40	**0.012**	1.23	0.74; 2.05	0.431	1.33	0.84; 2.09	0.220
Commercial confectionery	0.94	0.61; 1.44	0.773	1.12	0.63; 1.97	0.708	1.70	1.05; 2.74	**0.031**
Home-made confectionery	0.73	0.53; 1.02	0.064	0.71	0.46; 1.09	0.119	1.43	1.02; 2.01	**0.039**
Fast food	0.96	0.68; 1.36	0.812	1.25	0.78; 2.01	0.347	1.91	1.28; 2.86	**0.002**
Frequency of snacking	0.94	0.74; 1.21	0.648	0.92	0.67; 1.26	0.605	1.41	1.08; 1.85	**0.012**
Home-cooked food	1.09	0.86; 1.38	0.489	0.75	0.56; 1.02	0.068	1.51	1.17; 1.95	**0.002**
Home-delivered food from catering establishments	1.40	0.99; 1.97	0.054	3.03	1.60; 5.71	**<0.001**	7.13	4.06; 12.51	**<0.001**
Home-delivered food from stores	1.48	1.06; 2.06	**0.021**	2.70	1.20; 6.10	**0.017**	17.37	8.71; 34.65	**<0.001**
**Increased physical activity**	2.45	1.67; 3.59	**<0.001**	0.77	0.44; 1.33	0.345	1.18	0.76; 1.83	0.468
**Increased body weight**	0.96	0.73; 1.26	0.768	0.94	0.68; 1.32	0.732	1.16	0.87; 1.54	0.326

* Odds ratios adjusted for sex, age and place of residence; ** Odds ratios adjusted for sex, age and education; statistically significant *p*-values are bolded; OR—odds ratio; CI—confidence interval.

**Table 4:** Odds ratios for decreased lifestyle habits and body weight during the COVID-19 quarantine by education and place of residence.

Variables	Education *			Place of Residence **					
	High Versus Low			Towns Versus Villages			Cities Versus Villages		
	OR	95% CI	*p*-Value	OR	95% CI	*p*-Value	OR	95% CI	*p*-Value
**Decreased food intake**									
Meat and meat products	1.65	1.05; 2.59	**0.031**	0.76	0.44; 1.31	0.324	0.53	0.32; 0.88	**0.013**
Fish and seafood	0.73	0.46; 1.17	0.192	0.58	0.34; 0.78	**0.040**	1.08	0.34; 0.85	**0.009**
Fresh fruits or berries	0.97	0.52; 1.82	0.928	0.50	0.24; 1.06	0.071	0.51	0.27; 0.96	**0.037**
Commercial confectionery	1.38	0.98; 1.95	0.067	0.58	0.39; 0.88	**0.010**	0.43	0.30; 0.63	**<0.001**
Home-made confectionery	2.09	1.36; 3.22	**<0.001**	1.02	0.67; 1.76	0.749	0.42	0.25; 0.71	**<0.001**
Chocolate and sweets	1.98	1.32; 2.98	**0.001**	0.70	0.44; 1.12	0.136	0.37	0.24; 0.60	**<0.001**
Sweetened drinks, lemonade	1.59	1.09; 2.31	**0.015**	0.76	0.48; 1.21	0.240	0.67	0.45; 1.02	0.060
Fast food	1.82	1.25; 2,64	**0.002**	0.75	0.48; 1.17	0.206	0.49	0.32; 0.74	**<0.001**
Home-cooked food	1.66	0.86; 3.17	0.130	2.14	1.06; 4.36	**0.035**	0.50	0.21; 1.17	0.111
**Decreased physical activity**	1.26	0.98; 1.60	0.068	0.96	0.71; 1.31	0.809	1.01	0.77; 1.32	0.933
**Decreased body weight**	1.42	0.89; 2.27	0.140	0.52	0.30; 0.92	**0.025**	0.45	0.27; 0.75	**0.002**

* Odds ratios adjusted for sex, age and place of residence; ** Odds ratios adjusted for sex, age and education; statistically significant *p*-values are bolded; OR—odds ratio; CI—confidence interval.

**Table 5:** Odds ratios * for changes in lifestyle habits and body weight during the COVID-19 quarantine by the characteristic of the same variables in the subsequent post-quarantine period.

Variables	OR	95% CI	*p*-Value	OR	95% CI	*p*-Value
**Increased food intake**	**A few days a week versus 1–4 times a month or less often**			**Daily versus 1–4 times a month or less often**		
Meat and meat products	1.34	0.84; 2.15	0.222	2.74	1.51; 4.96	**0.001**
Fish and seafood	2.02	1.20; 3.40	**<0.001**	6.73	2.96; 15.28	**<0.001**
Milk and milk products	2.06	0.94; 4.51	0.072	2.17	1.00; 4.70	**0.049**
Fresh vegetables	1.13	0.39; 3.31	0.822	2.93	1.05; 8.16	**0.040**
Fresh fruits or berries	1.17	0.54; 2.55	0.685	3.01	1.48; 6.09	**0.002**
Commercial confectionery	1.57	1.00; 2.45	**0.048**	4.10	2.41; 6.88	**<0.001**
Chocolate and sweets	2.52	1.67; 3.80	**<0.001**	3.21	1.95; 5.29	**<0.001**
Sweetened drinks, lemonade	3.49	2.11; 5.77	**<0.001**	5.75	3.15; 10.51	**<0.001**
Fast food	2.53	1.73; 3.71	**<0.001**	5.76	2.68; 12.35	**<0.001**
Frequency of snacking	1.19	0.868; 1.63	0.283	2.70	1.32; 5.54	**0.007**
**Decreased food intake**	**A few days a week versus 1–4 times a month or less often**			**Daily versus 1–4 times a month or less often**		
Milk and milk products	0.64	0.36; 1.16	0.146	0.164	0.74; 0.37	**<0.001**
Fresh vegetables	0.38	0.15; 0.95	0.039	0.154	0.06; 0.41	**<0.001**
Fresh fruits or berries	0.64	0.33; 1.22	0.176	0.198	0.09; 0.43	**<0.001**
Commercial confectionery	0.73	0.52; 1.01	0.060	0.243	0.11; 0.57	**0.001**
Chocolate and sweets	0.50	0.32; 0.76	**0.001**	0.337	0.16; 0.71	**0.004**
Sweetened drinks, lemonade	0.81	0.51; 1.17	0.349	0.224	0.07; 0.72	**0.012**
**Increased physical activity**	**1–3 times a week versus less often than once a week**			**At least 4 times a week versus less often than once a week**		
	4.31	2.23; 8.33	**<0.001**	5.73	2.93; 11.22	**<0.001**
**Decreased physical activity**	1.43	1.09; 1.87	**0.009**	0.81	0.60; 1.11	0.189
**Increased body weight**	**Overweight versus low or normal weight**			**Obesity versus low or normal weight**		
	2.74	2.04; 3.67	**<0.001**	3.05	2.08; 4.47	**<0.001**
**Decreased body weight**	1.17	0.61;2.26	0.644	1.10	0.67; 1.80	0.698

* Odds ratios adjusted for sex, age, education and place of residence; statistically significant *p*-values are bolded; OR—odds ratio; CI—confidence interval.

**Table 6:** The proportion (%) of respondents whose changes in lifestyle habits and body weight during the COVID-19 quarantine persisted in the post-quarantine period.

Variables	Persisted Increased Lifestyle Habits and Body Weight (%)	Persisted Decreased Lifestyle Habits and Body Weight (%)
**Food intake**		
Meat and its products	37.4	51.6
Fish and seafood	61.4	43.3
Milk and milk products	58.6	45.6
Cereal products	69.9	38.6
Fresh vegetables	70.7	60.0
Fresh fruits or berries	65.9	58.5
Commercial confectionery	32.7	51.4
Home-made confectionery	28.0	59.2
Chocolate and sweets	33.1	57.8
Sweetened drinks, lemonade	33.3	71.4
Fast food	28.7	48.9
Amount of food eaten	27.4	31.9
Frequency of snacking	23.2	34.2
Home-cooked food	38.5	38.6
Home-delivered food from catering establishments	22.3	58.0
Home-delivered food from stores	40.9	59.9
**Physical activity**	54.3	30.0
**Body weight**	64.3	56.2

**Table 7:** Odds ratios * for the persistence of changes in lifestyle habits and body weight in the subsequent post-quarantine period by education and place of residence.

Variables	Education *			Place of Residence **					
	High Versus Low			Towns Versus Villages			Cities Versus Villages		
	OR	95% CI	*p*-Value	OR	95% CI	*p*-Value	OR	95% CI	*p*-Value
**Increased food intake**									
Fish and seafood	1.249	0.43; 3.61	0.681	2.244	0.51; 9.85	0.284	5.558	1.37; 22.55	**0.016**
Fresh vegetables	0.977	0.44; 2.17	0.954	0.214	0.07; 0.66	**0.008**	0.337	0.12; 0.97	**0.043**
Home-made confectionery	0.900	0.45; 1.92	0.768	0.742	0.32; 1.78	0.503	0.439	0.22; 0.88	**0.021**
Home-delivered food from stores	1.267	0.70; 2.29	0.434	0.155	0.03; 0.97	**0.047**	0.632	0.16; 2.52	0.516
**Decreased food intake**									
Fresh fruits or berries	0.103	0.02; 0.48	**0.004**	2.655	0.43; 16.42	0.293	3.228	0.66; 15.82	0.148
Amount of food eaten	0.350	0.13; 0.93	**0.035**	1.845	0.55; 6.33	0.324	1.118	0.37; 3.40	0.844
**Increased physical activity**	0.798	0.35; 1.84	0.597	0.505	0.16; 1.65	0.257	0.758	0.29; 1.97	0.569
**Decreased physical activity**	0.666	0.41; 1.08	0.097	0.839	0.49; 1.45	0.526	0.442	0.26; 0.74	**0.002**
**Increased body weight**	0.543	0.33; 0.89	**0.017**	1.077	0.57; 2.04	0.819	1.147	0.66; 2.00	0.629
**Decreased body weight**	0.426	0.17; 1.08	0.073	1.774	0.55; 5.76	0.340	2.321	0.82; 6.57	0.113

* Odds ratios adjusted for sex, age and place of residence; ** Odds ratios adjusted for sex, age and education; statistically significant *p*-values are bolded; OR—odds ratio; CI—confidence interval.

**Table 8:** Distribution (%) of respondents by lifestyle habits and body weight after COVID-19 quarantine.

Variables	N	%	N	%	N	%
**Food intake**	**Daily**		**A few days a week**		**1–4 times a month or less often**	
Red meat	386	25.8	769	51.3	344	22.9
Chicken	233	15.5	1008	67.2	258	17.3
Processed meat products	166	11.1	707	47.2	624	41.7
Fish and seafood	52	3.5	589	39.3	858	57.2
Milk and milk products	665	44.3	564	37.6	271	18.1
Cereal products	344	22.9	522	34.8	633	42.3
Legumes	64	4.3	428	28.6	1006	67.2
Nuts	230	15.4	524	35.0	743	49.6
Fresh vegetables	885	59.1	524	35.0	88	5.9
Fresh fruits or berries	755	50.4	528	35.2	216	14.4
Confectionery	150	10.0	565	37.7	783	52.3
Chocolate and sweets	203	13.6	564	37.7	730	48.7
Sweetened drinks, lemonade	118	7.9	313	20.9	1069	71.2
Fast food	31	2.1	245	16.3	1224	81.6
Unhealthy snacks	32	2.2	242	16.1	1226	81.7
**Physical activity**	**4 times a week or more often**		**1–3 times a week**		**Less often than once a week**	
	417	27.8	595	39.7	488	32.5
**Body weight**	**BMI < 25**		**BMI 25–29.9**		**BMI ≥ 30**	
	711	47.7	582	39.0	200	13.4

**Table 9:** Odds ratios for some lifestyle habits and overweight in the post-quarantine period by education and place of residence.

Variables	Education *			Place of Residence **					
	High Versus Low			Towns Versus Villages			Cities Versus Villages		
	OR	95% CI	*p*-Value	OR	95% CI	*p*-Value	OR	95% CI	*p*-Value
Red meat daily	0.635	0.49; 0.83	**0.001**	1.046	0.75; 1.45	0.788	1.437	1.08; 1.91	**0.013**
Poultry daily	0.893	0.66; 1.22	0.478	1.488	0.95; 2.34	0.089	3.340	2.28; 4.89	**<0.001**
Meat products daily or several times a week	0.544	0.43; 0.69	**<0.001**	0.753	0.56; 1.01	0.056	0.676	0.52; 0.88	**0.003**
Fish and seafood daily or several times a week	1.701	1.35; 2.14	**<0.001**	1.056	0.80; 1.40	0.705	1.451	1.13; 1.86	**0.003**
Milk and milk products daily	1.409	1.12; 1.77	**0.003**	0.708	0.54; 0.94	**0.016**	0.956	0.75; 1.22	0.722
Porridge daily	1.603	1.23; 2.08	**<0.001**	0.548	0.38; 0.79	**0.001**	1.088	0.82; 1.45	0.562
Legumes daily or several times a week	1.664	1.31; 2.11	**<0.001**	0.720	0.53; 0.98	**0.034**	1.058	0.82; 1.37	0.671
Nuts daily or several times a week	1.970	1.56; 2.49	**<0.001**	0.711	0.54; 0.94	**0.018**	1.417	1.11; 1.82	**0.006**
Fresh vegetables daily	1.885	1.48; 2.40	**<0.001**	1.039	0.79; 1.37	0.789	1.533	1.19; 1.97	**0.001**
Fresh fruits or berries daily	1.519	1.21; 1.91	**<0.001**	0.911	0.69; 1.20	0.510	1.751	1.37; 2.24	**<0.001**
Confectionery daily or several times a week	0.902	0.72; 1.13	0.377	0.672	0.51; 0.89	**0.005**	1.087	0.85; 1.39	0.506
Sweetened drinks daily or several times a week	0.572	0.44; 0.75	**<0.001**	0.587	0.43; 0.81	**0.001**	0.680	0.52; 0.90	**0.006**
Fast food daily or several times a week	0.581	0.42; 0.81	**0.001**	0.673	0.45; 1.00	0.051	1.122	0.81; 1.56	0.496
Unhealthy snacks daily or several times a week	0.678	0.49; 0.94	**0.018**	0.576	0.39; 0.85	**0.005**	0.751	0.54; 1.05	0.089
Physical activity at least 4 times a week	1.237	0.96; 1.59	0.097	0.729	0.54; 0.99	**0.044**	0.773	0.59; 1.01	0.061
Overweight (BMI ≥ 25 kg/m^2^)	0.740	0.58–0.94	**0.014**	1.114	0.83–1.49	0.471	0.758	0.59–0.98	**0.037**

* Odds ratios adjusted for sex, age and place of residence; ** Odds ratios adjusted for sex, age and education; statistically significant *p*-values are bolded. OR—odds ratio; CI—confidence interval.

## Data Availability

The data presented in this study are available on request from the corresponding author. The data are not publicly available due to ethical issues.
